# A four-stage DEA-based efficiency evaluation of public hospitals in China after the implementation of new medical reforms

**DOI:** 10.1371/journal.pone.0203780

**Published:** 2018-10-03

**Authors:** Wanhui Zheng, Hong Sun, Peilin Zhang, Guojiang Zhou, Quanyu Jin, Xiaoqin Lu

**Affiliations:** 1 Xiangya School of Public Health, Central South University, Changsha, P. R., China; 2 Hospital Cost Management Research Center, The Ninth People’s Hospital of Chongqing, Chongqing, P. R., China; 3 Xiangya Hospital, Central South University, Changsha, P. R., China; 4 Department of Human Resource and Performance Appraisal, Shanghai Skin Disease Hospital, Shanghai, P. R., China; 5 Yubei Maternity & Child Healthcare Hospital, Chongqing, P. R., China; Chongqing Medical University, CHINA

## Abstract

This study applied the non-parametric four-stage data envelopment analysis method (Four-Stage DEA) to measure the relative efficiencies of Chinese public hospitals from 2010 to 2016, and to determine how efficiencies were affected by eight factors. A sample of public hospitals (n = 84) was selected from Chongqing, China, including general hospitals and traditional Chinese medicine hospitals graded level 2 or above. The Four-Stage-DEA method was chosen since it enables the control of the impact of environment factors on efficiency evaluation results. Data on the number of staff, government financial subsidies, the number of beds and fixed assets were used as input whereas the number of out-patients and emergency department patients and visits, the number of discharged patients, medical and health service income and hospital bed utilization rate were chosen as study outputs. As relevant environmental variables, we selected GDP per capita, permanent population, population density, number of hospitals and number of available sickbeds in local medical institutions. The relative efficiencies (i.e. technical, pure technical, scale) of sample hospitals were also calculated to analyze the change between the first stage and fourth stage every year. The study found that Four-Stage-DEA can effectively filter the impact of environmental factors on evaluation results, which sets it apart from other models commonly used in existing studies.

## Introduction

In March 2009, the Chinese government formally launched healthcare reform as a long-term goal. China's investment in health infrastructure has increased significantly, resulting in the primary medical and health service systems being strengthened. Accessible medical insurance coverage has been achieved in a relatively short period of time and basic medical care and health services are available to all. In addition, the proportion of total health costs covered by patients has decreased, and the equalization of basic public health services has continued to advance. Subsequently, rates of child and maternal mortality and infectious disease morbidity have been significantly reduced, and health levels and life expectancies of Chinese residents have been significantly improved.

As is the case with many other countries, China faces many challenges in the reform and development of its medical and health services. Chronic diseases such as cancer, diabetes and heart diseases have become major health threats with aging of the population. Moreover, according to an important study, total health expenditure (THE) in China is expected to increase from 3.53 trillion yuan in 2014 to 15.805 trillion yuan in 2035, with a phased average annual growth rate of 8.4%. More importantly, health expenditure as a proportion of GDP will rise from 5.6% in 2014 to more than 9% in 2035, and incremental health expenditure of more than 60% was due to the increase in hospital services [1].Therefore, efficiency evaluation should pay attention to hospital efficiency, especially public hospitals above level II. Because that China's health institutions can be designated are Primary, Secondary or Tertiary institutions based on their ability to provide medical care, medical education and conduct medical research. A primary hospital is typically a township hospital that contains less than 100 beds. They are tasked with providing preventive care, minimal health care and rehabilitation services. Secondary hospitals tend to be affiliated with a medium size city, county or district and contain more than 100 beds, but less than 500. They are responsible for providing comprehensive health services, as well as medical education and conducting research on a regional basis. Tertiary hospitals round up the list as comprehensive or general hospitals at the city, provincial or national level with a bed capacity exceeding 500. They are responsible for providing specialist health services, perform a bigger role with regard to medical education and scientific research and they serve as medical hubs providing care to multiple regions [2]. According to economic type or sources of funding, Chinese hospitals can also be divided into public hospitals and private hospitals and the former are the most important component of health systems and account for a large share of health expenditure in China.

The evaluation of the effectiveness of public hospital reform is very important and beneficial since it is of benefit to the government and hospital managers to facilitate an understanding of the current situation of the reforming processes and to allow for targeted measures. The process can help measure the effects of reform of public hospitals in China, can assist in selecting an appropriate evaluation method and indicators and how to control or eliminate the influence of environmental factors on the outcome. Data enveloping analysis (DEA) is a multiple-input multiple-output nonparametric evaluation method and has already been widely employed to estimate the relative efficiencies of public hospitals, particularly over recent years, including measures of overall efficiency and technical efficiency, pure technical efficiency and scale efficiency. The Four-Stage-DEA method [3–5] is mostly based on the common variants of DEA (e.g., C^2^R, BC^2^ and Malmquist) which have been used in some previous studies as three-stage-DEA [6–8]. Most of those studies were focused on analyzing the influence of performance metrics on results and the external factors were first incorporated by Zhang and Liu (2013) [9]. However, previous studies failed to effectively translate into decision making practice because environmental variables were not effectively eliminated or controlled. Since the objectivity of evaluation results can be questioned, it is doubtful that past evaluation results can be adopted by government regulators and hospital managers.

## Materials and methods

### Data source

To assist medical institutions in keeping track of information on health resources, the Chongqing Health and Family Planning Commission collects data from city- and county-level medical institutions and hospitals on an annual basis, compiles the data into the Collection of Major Metrics of Some Medical Institutions in Chongqing and releases these data to the city’s medical agencies. In the present study, the input-output data were derived from the collection released during 2010–2016, along with information published on the official websites of the Chongqing Health Information Center and 84 public hospitals. The data for environmental variable were acquired from the Chongqing Statistical Yearbooks of years 2011–2017. To guarantee homogeneity of the evaluated hospitals, only comprehensive hospitals and traditional Chinese hospitals graded level 2 or above under the Chinese hospital criterion were selected. An additional reason behind this selection is that according to a previous investigation, over 80% of the traditional Chinese hospitals now adopt Western medical technologies. Hospitals with incomplete data over the study period were excluded; notable examples were the People’s Hospital in Gaoxin district and University-Town Hospital of Chongqing Medical University. In addition, the hospitals that are governed by a different administrative area, such as the Shuangqiao and Wansheng hospitals, were also excluded. A total of 84 hospitals that satisfied all the inclusion criteria were selected, and sorted according to the Collection of 2016. These hospitals were sequentially labeled H1 to H84.

### Methodology

The input-output metrics and the environmental variable were descriptively analyzed using Excel 2010. A database was constructed in compliance with the requirements of Deap2.1 and Four-Stage DEA was used for analysis.

Stage 1: “Coarse” efficiency calculation. With Deap2.1, the DEA value of the 84 hospitals and the slackness of each input were computed using the input-oriented DEA-BC^2^ model. The means of integrated efficiency, pure technical efficiency and scale efficiency, along with tendency were then analyzed. Finally, the frequency distribution of hospital efficiencies was determined (100%, 80–99.9%, 60–79.9%, < 60% [10]).

Stage 2: Tobit regression analysis. With the input relaxation obtained from Stage 1 as the dependent variable and the environmental factor as the independent variable, I Tobit regression models were constructed, where I denotes the number of input elements. A Tobit truncated model-based analysis was performed via stata14. The regression model is:

$$S_{ik}=\alpha_{i}+\beta_{i}Z_{ik}+u_{i}(i=1,2,\ldots \;I;k=1,2,\ldots ,\;K)$$ (1)

Where

$$S_{ik}=(1-{\hat{\theta }}_{k})x_{ik}+S_{ik}^{-}$$ (2)

denotes the total relaxation of the i-th input element obtained from the classic DEA method,*Z*_*ik*_denotes the vector of exogenous environmental variables,*α*_*i*_ denotes the constant and *β*_*i*_ denotes the vector coefficients which need to be estimated.*U*_*i*_ denotes error.

Stage 3: The original input quantities were jointly adjusted to the regression result of the second step and the maximum fitted value using Excel 2010. The method can be formulated as:

$$x_{ik}^{adj}=x_{ik}+\{\max^{k}[{\hat{S}}_{ik}]-{\hat{S}}_{ik}\}i=1,2,\ldots ,I,k=1,2,\ldots ,K)$$ (3)

Where

$$\max^{k}[{\hat{S}}_{ik}]-{\hat{S}}_{ik}=0$$ (4)

indicates that the input quantity is not adjusted.

And

$$\max^{k}[{\hat{S}}_{ik}]-{\hat{S}}_{ik}>0$$ (5)

means that given a congenial external environment, the original input causes a decrease in DEA efficiency. This can be attributed to the fact that the largest fitted value indicates that the decision-making unit is under the worst external environment currently. Adjusting and improving the external environment can reduce the high efficiency that is obtained from the congenial factors [4, 11].

Stage 4: The adjusted value of relative efficiency was computed jointly using the adjusted input quantity, original output, Deap2.1 and DEA—BC^2^. The efficiency and frequency distributions of hospital efficiency were compared with those of the first stage.

After the four stages were completed, the influence of the environmental factors on efficiency could be effectively eliminated or alleviated, making the evaluation result more objective.

### Selection of metrics

Input/output metrics and environmental variables for DEA analysis

Considering the fact that the 84 public hospitals are rated above level 2, biblio-metric analysis and our previous quantitative research were performed to determine the input/output metrics and the eight external environmental variables chosen for the current study [12–17]. Their definitions and data sources are given in Table 1.

**Table 1:** Definitions and data source of input/output metrics and environmental variables.

Category	Variable	Definition	Data source ^a^
**Input**	**Number of staff**	Registered on-the-job staff of the hospital at the end of each year (medical technicians, other technicians, managers and logistics workers, etc.)	The size of manpower at the end of each year = medical service revenue/revenue generated by each worker
	**Government financial subsidies** (10,000 RMB)	The total financial and business funds (including fixed and targeted grants) obtained by the hospital from its supervising authorities or sponsors	Financial subsidy = total revenue × the proportion of financial subsidy in hospital revenue
	**Number of beds**	The actual and fixed number of hospital sickbeds (those not occupied) at year end, including regular sickbeds, bunk beds, monitoring beds, sickbeds under disinfection and repair and out-of-service sickbeds due to expansion or major renovation.	Direct extraction
	**Fixed assets** (10,000 RMB)	Tangible assets that have been used for more than one year and whose amount and specific standards have not changed.	Fixed assets = medical revenue/(revenue per 100 yuan of fixed assets/100)
**Output**	**Number of out-patients and emergency department patients and visits** (10,000 persons)	Number of out-patient (emergency) cases at year end	Direct extraction
	**Number of discharged patients** (10,000 persons)	Number of in-hospitalization cases at the end of each year	Direct extraction
	**Medical and health service income** (10,000 RMB)	Revenue obtained by a medical and healthcare organization during its operations, including incomes from patient occupancy, diagnosis, examination, treatment, surgery, laboratory tests, nursing and other income.	Direct extraction
	**Hospital bed utilization rate (**%**)**	The percentage occupied sickbeds per day	Direct extraction
**Environmental variable**	**GDP per capita** (RMB)	Per-capita GDP of the area where the hospital is located	Direct extraction (2011–2016), Calculation (2010)
	**Permanent population** (10,000 persons)	Permanent population in the area where the hospital is located	Direct extraction
	**Population density** (persons/km^2^)	Number of permanent residents in the area where the hospital is located	Population density = number of permanent residents/the size of the area
	**Number of hospitals** (per 10,000 people)	Number of hospitals per 10,000 persons	Total number of hospitals = total number of hospitals/permanent population in the area
	**Number of available sickbeds in local medical institutions**	The actual and fixed number of sickbeds (those that have not been occupied) in the area at year end	Direct extraction
	**Number of medical staff in local regions**	The professional medical staff such as practicing physicians, practicing assistant physicians, registered nurses, pharmacists, laboratory technicians, imaging technicians, health supervisors and trainees (pharmacists, nurses and technicians)	Direct extraction
	**Licensed and assistant doctors in local regions**	Doctors rated whose as “practicing physicians” or “practicing assistant physicians" and who are engaged in medical treatment and preventive healthcare work	Direct extraction
	**Licensed and assistant nurses in local regions**	Nurses who have a registered nurse certificate and are engaged in practical nursing work	Direct extraction

^a^ All input-output data are extracted or calculated from the "Collection of Indicators of Some Medical Institutions in Chongqing" (2010–2016); All data of environmental variables are extracted or calculated from the "Chongqing Statistical Yearbook" (2011–2017).

### Descriptive analysis of input/output metrics and environmental variables

Input/output metrics and environmental variables were first descriptively analyzed before evaluating relative efficiency via Deap2.1.

Analysis of input/output metrics: Excluding sickbed utilization, the mean of the remaining seven metrics increased for seven years consecutively. Excluding financial investment and sickbed utilization, the maximum values of the remaining six metrics also increased on an annual basis. The minimum value of five metrics, excluding manpower, financial subsidy and sickbed utilization, increased annually, as shown in Table 2.

**Table 2:** Descriptive statistics of inputs/outputs variables from 2010 to 2016.

			2010	2011	2012	2013	2014	2015	2016
**Invest**	**Number of staf**f	**Mean**	387.21	406.90	589.75	679.81	719.51	811.71	904.26
		**Maximum**	2 621.38	2 778.25	4 418.26	4 907.64	5 353.31	5 840.91	6 266.80
		**Minimum**	35.83	37.01	37.00	77.01	43.00	44.00	106.98
		**Standard deviation**	368.12	385.00	709.60	694.78	762.36	813.25	870.63
	**Government financial subsidies** (10,000 RMB)	**Mean**	1 068.42	1 820.08	1 823.79	1 760.26	2 104.11	3 094.30	3 246.88
		**Maximum**	6 561.93	11 721.50	11 948.53	8 417.53	10 782.20	15 762.41	16 541.26
		**Minimum**	19.98	100.51	47.35	0.00	39.79	110.73	134.89
		**Standard deviation**	1 239.52	2 451.33	2 218.92	1 829.71	2 205.88	2 975.62	3 316.84
	**Number of beds**	**Mean**	438.05	496.68	575.25	627.93	678.96	712.25	757.21
		**Maximum**	2 511.00	2 949.00	3 000.00	3 321.00	3 328.00	3 439.00	3 585.00
		**Minimum**	30.00	60.00	60.00	60.00	80.00	95.00	100.00
		**Standard deviation**	396.25	453.50	507.06	536.35	537.38	559.17	577.44
	**Fixed assets** (10,000 RMB)	**Mean**	17 797.92	21 191.85	25 630.41	29 463.40	32 914.28	37 595.90	44 648.00
		**Maximum**	162 802.14	165 140.77	186 647.53	249 813.67	273 471.49	379 865.34	480 077.16
		**Minimum**	595.45	642.13	937.36	1 308.03	1 120.28	1 077.93	1 636.84
		**Standard deviation**	24 677.06	27 320.17	36 599.33	41 986.85	46 293.58	55 094.37	67 226.42
**Output**	**Number of out-patient and emergency department patients and visits** (10,000 persons)	**Mean**	25.84	29.18	34.45	35.92	41.77	45.12	47.93
		**Maximum**	183.80	202.55	215.13	258.63	289.75	306.35	325.74
		**Minimum**	1.83	2.17	2.69	3.07	3.02	3.58	3.60
		**Standard deviation**	26.37	31.45	38.56	40.31	44.80	47.50	51.12
	**Number of discharged patients** (10,000 persons)	**Mean**	1.47	1.67	1.99	2.18	2.47	2.64	2.90
		**Maximum**	7.82	8.83	9.76	11.12	12.09	12.83	14.06
		**Minimum**	0.07	0.10	0.14	0.14	0.16	0.18	0.18
		**Standard deviation**	1.25	1.42	1.63	1.72	1.92	2.00	2.17
	**medical and health service income** (10,000 RMB)	**Mean**	14 306.49	17 487.91	23 009.82	27 678.01	33 037.16	37 089.90	41 304.91
		**Maximum**	192 318.17	225 730.92	27 2562.73	328 370.48	390 845.45	430 007.56	475 900.49
		**Minimum**	366.40	632.07	1 073.50	1 192.87	1 392.85	1 659.26	1 950.17
		**Standard deviation**	24 299.13	28 848.75	36 678.51	43 072.54	51 150.89	56 165.40	61 788.33
	**Hospital bed utilization rate (**%**)**	**Mean**	92.77	95.60	95.50	94.05	92.79	91.92	90.81
		**Maximum**	157.20	149.04	136.61	131.73	129.76	126.28	118.24
		**Minimum**	36.10	43.99	54.79	52.41	47.67	49.80	50.58
		**Standard deviation**	17.51	17.74	14.43	14.17	14.15	13.25	11.67

Analysis of environmental variables: Excluding hospital density, the mean and maximum values of the remaining seven metrics increased annually, and excluding hospital and population density, the maximum values of the remaining six metrics also increased on an annual basis. whereas the minimal values of the permanent population and population density decreased. This can be attributed to the fact that this city’s population is concentrated in the major downtown and economically-developed regions, resulting in annual decreases in populations of remote counties. The minimum value of hospital density varied irregularly, whereas the minimum value of the remaining five metrics increased on an annual basis, as shown in Table 3.

**Table 3:** Descriptive statistics of environment variables from 2010 to 2016.

		2010	2011	2012	2013	2014	2015	2016
**GDP per capita (RMB)**	**Mean**	29 882.11	37 034.36	40 724.85	43 842.95	48 427.88	52 463.29	56 860.10
	**Maximum**	87 768.12	104 844.00	118 921.00	123 771.00	133 588.00	147 423.00	160 743.00
	**Minimum**	9 079.01	11 484.00	13 055.00	15 018.00	16 889.00	18 741.00	21 120.00
	**Standard deviation**	20 671.34	24 230.61	26 950.83	27 537.29	29 471.21	32 493.72	34 892.84
**Permanent population (10,000 persons)**	**Mean**	77.33	79.04	79.76	80.63	81.03	81.71	82.61
	**Maximum**	156.31	157.22	158.31	159.54	160.46	160.74	162.33
	**Minimum**	19.30	19.03	19.30	19.06	18.83	18.63	18.49
	**Standard deviation**	30.52	31.51	32.09	32.88	33.34	34.05	34.83
**Population density (persons/km**^**2**^**)**	**Mean**	2 592.64	2 641.09	2 688.10	2 703.09	2 708.72	2 715.24	2 748.66
	**Maximum**	27 395.65	27 782.61	28 230.43	28 269.57	28 278.26	28 239.13	28 573.91
	**Minimum**	58.68	57.86	58.68	57.95	57.25	56.64	56.22
	**Standard deviation**	6 968.56	7 066.75	7 181.79	7 190.54	7 192.63	7 181.87	7 267.01
**Number of hospitals (per 10,000 persons)**	**Mean**	0.54	0.52	0.52	0.55	0.56	0.57	0.58
	**Maximum**	1.45	1.37	1.35	1.66	1.38	1.38	1.41
	**Minimum**	0.29	0.26	0.28	0.28	0.29	0.30	0.29
	**Standard deviation**	0.25	0.22	0.22	0.28	0.23	0.23	0.24
**Number of available sickbeds in local medical institutions**	**Mean**	3 066.36	3 463.50	3 901.87	4 359.85	4 752.65	5 160.92	5 645.40
	**Maximum**	8 112.00	8 835.00	9 423.00	9 960.00	10 356.00	10 838.00	12 120.00
	**Minimum**	669.00	815.00	818.00	840.00	910.00	912.00	872.00
	**Standard deviation**	1 867.81	2 087.29	2 310.06	2 530.25	2 629.16	2 748.49	2 915.64
**Number of medical staff in local regions**	**Mean**	3 281.05	3 739.12	4 085.04	4 407.99	4 845.42	5 166.89	5 609.81
	**Maximum**	10 555.00	12 000.00	12 871.00	13 768.00	15 373.00	16 500.00	17 455.00
	**Minimum**	549.00	616.00	644.00	705.00	707.00	776.00	829.00
	**Standard deviation**	2 534.65	2 856.05	3 092.11	3 316.08	3 677.84	3 932.24	4 146.01
**Licensed and assistant doctors in local regions**	**Mean**	1 360.42	1 519.31	1 584.02	1 684.89	1 849.42	1 870.04	1 996.64
	**Maximum**	4 047.00	4 399.00	4 449.00	4 788.00	5 724.00	5 564.00	5 602.00
	**Minimum**	217.00	235.00	234.00	260.00	266.00	310.00	295.00
	**Standard deviation**	978.42	1 079.43	1 103.31	1 191.61	1 412.77	1 357.08	1 382.91
**Licensed and assistant nurses in local regions**	**Mean**	1 198.04	1 359.95	1 621.43	1 780.49	2 017.25	2 222.31	2 471.04
	**Maximum**	4 525.00	5 465.00	6 285.00	6 532.00	7 313.00	7 983.00	8 424.00
	**Minimum**	77.00	98.00	144.00	178.00	182.00	218.00	253.00
	**Standard deviation**	1 114.45	1 343.87	1 530.81	1 592.70	1 779.19	1 936.29	2 032.74

## Empirical results

### Results of DEA analysis for Stage 1

Table 4 shows the relative efficiency and frequency distribution of the 84 public hospitals in Chongqing. It can be seen that although the mean of relative efficiency of the samples was high (> 0.862), the technical, pure technical and scale efficiencies displayed different characteristics.

**Table 4:** Relative efficiency and frequency distribution of sampled hospitals at Stage 1 from 2010 to 2016.

		2010	2011	2012	2013	2014	2015	2016
**Technical efficiency** ^a^	**Mean**	0.862	0.864	0.876	0.884	0.880	0.876	0.890
	**Maximum**	1.000	1.000	1.000	1.000	1.000	1.000	1.000
	**Minimum**	0.478	0.486	0.535	0.427	0.503	0.503	0.492
	**Standard deviation**	0.150	0.136	0.130	0.127	0.124	0.124	0.115
	**Hospital ranking**							
	**100%**	26	24	30	30	27	25	26
	**80**–**99.9%**	34	36	30	36	38	35	41
	**60**–**79.9%**	19	23	21	16	17	21	15
	**<60%**	5	1	3	2	2	3	2
**Pure technical efficiency** ^b^	**Mean**	0.882	0.898	0.913	0.922	0.913	0.899	0.913
	**Maximum**	1.000	1.000	1.000	1.000	1.000	1.000	1.000
	**Minimum**	0.538	0.621	0.616	0.616	0.653	0.646	0.703
	**Standard deviation**	0.135	0.115	0.107	0.096	0.098	0.109	0.089
	**Hospital ranking**							
	**100%**	30	34	39	39	32	30	29
	**80**–**99.9%**	31	30	30	35	39	36	44
	**60**–**79.9%**	20	20	15	10	13	18	11
	**<60%**	3	0	0	0	0	0	0
**Scale efficiency** ^c^	**Mean**	0.975	0.960	0.957	0.956	0.961	0.972	0.972
	**Maximum**	1.000	1.000	1.000	1.000	1.000	1.000	1.000
	**Minimum**	0.689	0.668	0.663	0.585	0.692	0.752	0.659
	**Standard deviation**	0.048	0.061	0.063	0.068	0.063	0.044	0.055
	**Hospital ranking**							
	**100%**	29	26	31	30	28	25	26
	**80**–**99.9%**	53	56	49	50	53	57	56
	**60**–**79.9%**	2	2	4	3	3	2	2
	**<60%**	0	0	0	1	0	0	0

^a^ Technical efficiency when the return to scale remains unchanged (comprehensive efficiency)

^b^ Pure technical efficiency when the return to scale is changing

^c^ Scale efficiency = CRS/VRS

#### Technical efficiency

Technical efficiency refers to the ability to comprehensively measure the distribution and utilization of resources in an evaluated hospital. As shown in Table 4, the maximum value was constantly 1.000 whereas others varied irregularly. The mean of technical efficiency of sampled hospitals increased during 2010–2013, decreased in 2014 and then rose again in 2016. The minimum value of technical efficiency peaked at 0.535 in 2012. In terms of the frequency distribution of hospital efficiency, relatively efficient hospitals (100%) accounted for about 1/3 of the total samples, whereas over 35% of the samples fell in the range of 80–99.9% and around 20% of the hospitals were in the range of 60–79.9%. H7 showed efficiencies consistently below 60%. These findings contrasted with the previous conclusion of annual increases for most metrics, prompting us to further explore the reasons behind relative inefficiency.

#### Pure technical efficiency

Pure technical efficiency is influenced by hospital management and technologies. It can be inferred from Table 4 that the pure technical efficiency of the samples was slightly higher than the technical efficiency for seven years consecutively, varying in a way similar to the technical efficiency. Note that for each of those seven years, the number of hospitals with relatively high pure technical efficiencies was consistently higher than the number of hospitals with relatively high technical efficiencies. No hospitals consistently showed pure technical efficiency values below 60% for a period of six consecutive years. The number of hospitals showing pure technical efficiencies in the range 80–99.9% or 60–79.9% varied irregularly. This finding also contradicts the previous conclusion of annual increases for most metrics.

#### Scale efficiency

Scale efficiency is influenced by hospital size. As shown in Table 4, the mean of scale efficiency fluctuated within a small range (0.956–0.975), and the minimum value fluctuated in the range of 0.585–0.752. For over 99.95% of the hospitals, the efficiencies were 100% or in the range of 80–99.9%. Only one hospital had an efficiency below 60% in 2013.

Considered together, Stage 1 analysis indicated that the mean technical efficiency of the 84 sampled hospitals was high, but the mean of scale efficiency was obviously higher than the mean of pure technical efficiency. This means that without considering external factors, the scale of sampled hospitals is suitable. Government departments and hospital management personnel should improve the relative efficiencies of hospitals by improving management and technology.

### Result of Tobit analysis for Stage 2

Using eight independent environmental variables and four input relaxation dependent variables, four Tobit models were constructed for regression analysis of the seven years. The basic interpretation of the analysis was that if the explanatory variable was directly proportional to the explained variable and the input slack variable, the environmental factor did not contribute to the relative efficiency of the hospital. On the other hand, if the explanatory variable was in reverse proportion to the explained variable and the input slack variable, that particular environmental factor contributed to the relative efficiency of the hospital. The observations have been summarized in Table 5:

**Table 5:** Regression analysis of environmental factors and input slack variables for Stage 2 from 2010 to2016.

Years	Explained variable	Constant term	Prob > chi2	Explanatory variable							
				GDP per capita (RMB)	Permanent population (10,000 persons)	Population density (persons / square kilometers)	Number of hospitals (for 10, 000 persons)	Number of available sickbeds in local medical institutions	Number of medical staff in local regions	Licensed and assistant doctors in local regions	Licensed and assistant nurses in local regions
**2010**	**The size of manpower at the end of each year**	112.64 (0.64)	0.44	0.00 (0.76)	−2.63 (0.37)	−0.02 (0.33)	−219.72 (0.42)	0.03 (0.54)	0.06 (0.41)	−0.24 (0.33)	0.11 (0.51)
	**Financial subsidy**	27.07 (0.85)	0.06	−0.00 (0.70)	0.41 (0.83)	0.00 (0.70)	−29.37 (0.79)	−0.04 (0.36)	−0.02 (0.69)	−0.12 (0.41)	0.26 (0.10)*
	**Number of available sickbeds**	−713.91 (0.64)	0.05	0.03 (0.26)	−3.22 (0.86)	−0.04 (0.68)	−1 218.34 (0.37)	0.79 (0.08)*	−0.40 (0.39)	−0.78 (0.57)	0.45 (0.75)
	**Fixed assets**	−153.45 (0.21)	0.01	0.00 (0.08)*	1.11 (0.39)	−0.00 (0.93)	−115.15 (0.35)	0.08 (0.02)**	−0.03 (0.44)	−0.02 (0.87)	−0.18 (0.12)
**2011**	**The size of manpower at the end of each year**	21.07 (0.00)***	0.00	0.00 (0.00)***	−2.39 (0.00)***	−0.04 (0.00)***	−182.52 (0.00)***	0.05 (0.00)***	−0.22 (0.00)***	0.56 (0.00)***	−0.14 (0.00)***
	**Financial subsidy**	6.49 (0.96)	0.27	−0.00 (0.70)	−0.83 (0.54)	−0.01 (0.17)	−57.34 (0.61)	−0.04 (0.25)	0.12 (0.21)	−0.13 (0.41)	0.02 (0.69)
	**Number of available sickbeds**	−5 968.56 (0.07)*	0.06	0.05 (0.28)	50.39 (0.12)	0.34 (0.19)	3 464.81 (0.17)	−2.27 (0.03)**	0.10 (0.97)	2.75 (0.55)	−1.05 (0.43)
	**Fixed assets**	−3.25 (0.99)	0.13	0.01 (0.32)	−5.40 (0.26)	−0.11 (0.55)	−135.78 (0.47)	0.19 (0.17)	−0.19 (0.60)	0.29 (0.62)	−0.20 (0.52)
**2012**	**The size of manpower at the end of each year**	−220.81 (0.00)***	0.00	0.00 (0.00)***	1.95 (0.00)***	0.01 (0.00)***	−49.09 (0.00)***	0.10 (0.00)***	−0.27 (0.00)***	−0.15 (0.00)***	0.32 (0.00)***
	**Financial subsidy**	−94.18 (0.53)	0.99	−0.00 (0.70)	1.42 (0.45)	0.00 (0.88)	10.84 (0.93)	0.01 (0.82)	−0.18 (0.39)	0.11 (0.65)	0.26 (0.32)
	**Number of available sickbeds**	−482.81 (0.89)	0.36	0.03 (0.55)	35.55 (0.45)	0.08 (0.80)	−1 579.45 (0.61)	0.47 (0.60)	−3.91 (0.41)	−2.31 (0.69)	7.55 (0.19)
	**Fixed assets**	−174.09 (0.47)	0.19	0.00 (0.31)	1.42 (0.66)	−0.06 (0.52)	−163.81 (0.50)	−0.09 (0.37)	0.26 (0.52)	−0.61 (0.25)	0.03 (0.95)
**2013**	**The size of manpower at the end of each year**	−20.96 (0.69)	0.82	0.00 (0.43)	−0.80 (0.40)	−0.02 (0.36)	−17.48 (0.67)	−0.01 (0.71)	−0.07 (0.62)	0.11 (0.56)	0.08 (0.65)
	**Financial subsidy**	278.53 (0.22)	0.29	−0.00 (0.25)	−4.92 (0.17)	−0.03 (0.10)*	−197.85 (0.22)	−0.08 (0.25)	0.56 (0.09)*	−0.56 (0.09)*	−0.44 (0.34)
	**Number of available sickbeds**	3 039.54 (0.27)	0.30	−0.05 (0.24)	−30.31 (0.42)	−0.46 (0.06)*	−1 893.41 (0.35)	−1.72 (0.06)*	−0.31 (0.93)	0.65 (0.86)	5.63 (0.30)
	**Fixed assets**	−90.92 (0.48)	0.81	0.00 (0.76)	−0.34 (0.86)	−0.06 (0.43)	13.05 (0.88)	0.00 (0.96)	−0.09 (0.61)	0.08 (0.66)	0.14 (0.53)
**2014**	**The size of manpower at the end of each year**	−16.18 (0.59)	0.12	0.00 (0.27)	−0.20 (0.59)	0.00 (0.37)	−29.90 (0.31)	0.01 (0.09^)^*	−0.01 (0.52)	−0.04 (0.28)	−0.03 (0.41)
	**Financial subsidy**	15.61 (0.93)	0.94	0.00 (0.64)	−0.92 (0.71)	−0.01 (0.68)	−136.00 (0.38)	−0.02 (0.61)	0.07 (0.56)	−0.06 (0.66)	−0.05 (0.79)
	**Number of available sickbeds**	−3 176.59 (0.32)	0.03	0.08 (0.07)*	−26.94 (0.54)	−0.19 (0.45)	77.72 (0.98)	−0.34 (0.69)	0.09 (0.96)	1.64 (0.05)**	−0.73 (0.85)
	**Fixed assets**	−201.22 (0.88)	0.35	0.00 (0.53)	1.47 (0.92)	−1.24 (0.47)	−521.35 (0.68)	0.18 (0.39)	0.38 (0.59)	−0.62 (0.54)	−0.88 (0.44)
**2015**	**The size of manpower at the end of each year**	−45.24 (0.32)	0.55	0.00 (0.86)	0.08 (0.86)	0.00 (0.39)	9.54 (0.78)	0.02 (0.18)	−0.03 (0.40)	−0.02 (0.65)	0.05 (0.34)
	**Financial subsidy**	−41.48 (0.88)	0.15	0.01 (0.07)*	−4.86 (0.19)	−0.05 (0.05)**	−308.81 (0.22)	−0.05 (0.53)	0.57 (0.09)*	−0.92 (0.03)**	−0.40 (0.31)
	**Number of available sickbeds**	−1 255.45 (0.70)	0.16	0.06 (0.16)	16.28 (0.67)	−0.14 (0.63)	−655.42 (0.81)	−0.88 (0.30)	−0.97 (0.80)	−1.66 (0.71)	3.86 (0.41)
	**Fixed assets**	−1 105.89 (0.01)***	0.00	0.01 (0.00)***	6.24 (0.23)	−0.13 (0.72)	404.86 (0.22)	0.18 (0.06)*	−0.39 (0.31)	0.14 (0.80)	0.21 (0.67)
**2016**	**The size of manpower at the end of each year**	−33.57 (0.18)	0.32	0.00 (0.36)	0.16 (0.45)	−0.01 (0.45)	13.87 (0.39)	0.00 (0.47)	0.00 (0.98)	−0.00 (0.83)	−0.01 (0.77)
	**Financial subsidy**	−57.46 (0.71)	0.17	0.00 (0.56)	−0.01 (0.99)	−0.20 (0.09)*	−66.73 (0.59)	−0.03 (0.41)	0.25 (0.08)*	−0.45 (0.01)***	−0.14 (0.48)
	**Number of available sickbeds**	−5 450.05 (0.22)	0.59	0.03 (0.49)	76.74 (0.08)*	0.57 (0.08)*	3 062.90 (0.32)	0.64 (0.41)	−5.21 (0.14)	0.77 (0.84)	6.70 (0.17)
	**Fixed assets**	−787.20 (0.04)**	0.01	0.00 (0.45)	5.66 (0.06)*	0.05 (0.04)**	378.74 (0.15)	0.12 (0.04)**	−0.37 (0.10)*	0.11 (0.57)	0.35 (0.20)

*Significant at the 0.10 level, two-tailed test

**Significant at the 0.05 level, two-tailed test

*** Significant at the 0.01 level,two-tailed test.

In the years 2010–2016, at the end of each year, manpower was directly proportional to the GDP per capita, meaning that increasing the hospital’s manpower was not conducive to the improvement of relative efficiency of hospitals.

The remaining variables were in either direct or reverse proportion to the environmental factors, implying that the contribution of each metric to an environmental factor varied with time.

Statistically significant regression results were only observed for some years.

After repeated verification of the original data, computed independently by two researchers, the above phenomenon persisted. On further consideration and inspired by studies in other fields, it was deemed absolutely necessary to alleviate or eliminate the influence of environmental factors on relative efficiency by fitting the original input.

### Result of fitting using Excel 2000 for Stage 3

Based on the Tobit-based regression analysis result of Stage 2, the largest fitting value was inserted in Table 6 to adjust the original input. This is because the largest fitting value is able to represent the worst external environment of the hospital, effectively alleviating the high efficiency that can be attributed to a congenial external environment. In this way, the hospitals can be assessed under similar circumstances to guarantee as much homogeneity as possible. In other words, the influence of environmental variables on efficiency evaluation can be considerably controlled or even eliminated. The results in Table 6 show that all metrics changed after the fitting process. Considerable increase in manpower, financial subsidy and sickbed availability was however seen only at the end of 2011. After repeatedly confirming that the original data and the calculation process were error-free, the results above were attributed to the statistical significance of the year (e.g., P = 0.00 for the manpower).

**Table 6:** Adjusted result of original input for Stage 3 from 2010 to 2016.

		2010	2011	2012	2013	2014	2015	2016
**Number of staff**	**Mean**	480.14	649.35	747.37	837.13	743.71	2 128.55	940.30
	**Maximum**	2 741.46	3 356.65	4 454.56	5 695.85	5 357.19	10 556.18	6 608.40
	**Minimum**	90.37	157.72	137.03	80.78	87.20	44.00	112.69
	**Standard deviation**	361.14	645.62	717.43	827.31	759.59	1 774.21	921.57
**Government financial subsidies** (10,000 RMB)	**Mean**	1 275.13	1 914.99	1 866.28	1 950.09	2 174.66	3 331.97	3 426.95
	**Maximum**	6 561.94	11 760.86	12 021.48	8 715.02	10 815.52	15 948.08	16 804.39
	**Minimum**	292.11	203.06	79.69	222.00	61.28	110.73	297.11
	**Standard deviation**	1 205.78	2 428.57	2 221.75	1 851.66	2 202.26	2 969.23	3 325.98
**Number of beds**	**Mean**	2 137.85	3 415.51	2 398.76	2 370.43	4 944.88	3 173.63	2 732.61
	**Maximum**	11 231.43	6 687.83	9 221.24	7 953.64	8 358.62	9 442.82	10 484.05
	**Minimum**	487.00	300.00	380.00	180.00	730.00	370.00	665.00
	**Standard deviation**	2 219.57	1 481.86	1 616.91	1 395.21	1 523.23	1 578.83	1 491.00
**Fixed assets** (10,000 RMB)	**Mean**	17 997.43	21 636.32	25 953.42	29 633.20	36 589.10	38 690.06	44 850.14
	**Maximum**	163 313.70	168 445.92	187 787.93	249 813.67	310 030.90	385 408.36	480 115.80
	**Minimum**	806.53	760.54	1 089.34	1 350.40	1 867.29	1 804.65	1 799.57
	**Standard deviation**	24 728.56	27 878.18	36 745.99	42178.15	51 716.46	55 750.86	67 198.94

### Result of DEA analysis for Stage 4

Table 7 compares the “net” relative efficiency with the “coarse” relative efficiency, which was analyzed from the perspectives of unchanged and changed variables, as described below.

**Table 7:** Comparison between the relative efficiencies calculated by Four-Stage-DEA during step1 and step 4 during 2010–2016.

		2010		2011		2012		2013		2014		2015		2016	
		Post-adjustment	Comparison with pre-adjustment results	Post-adjustment	Comparison with pre-adjustment results	Post-adjustment	Comparison with pre-adjustment results	Post-adjustment	Comparison with pre-adjustment results	Post-adjustment	Comparison with pre-adjustment results	Post-adjustment	Comparison with pre-adjustment results	Post-adjustment	Comparison with pre-adjustment results
**Technical efficiency**	**Mean**	0.862	−0.023	0.824	−0.040	0.813	−0.063	0.861	−0.023	0.884	0.004	0.832	−0.044	0.849	−0.041
	**Maximum**	1.000	0	1.000	0	1.000	0	1.000	0	1.000	0	1.000	0	1.000	0
	**Minimum**	0.478	−0.028	0.298	−0.188	0.353	−0.182	0.436	0.009	0.447	−0.056	0.231	−0.272	0.419	−0.073
	**Standard deviation**	0.150	0.025	0.171	0.035	0.163	0.033	0.149	0.022	0.141	0.017	0.165	0.041	0.143	0.028
	**Hospital ranking**														
	**100%**	26	2	22	−2	24	−6	29	−1	36	9	22	−3	22	−4
	**80–99.9%**	34	−9	31	−5	22	−8	30	−6	28	−10	28	−7	31	−10
	**60–79.9%**	19	4	22	−1	29	8	19	3	17	0	26	5	27	12
	**<60%**	5	3	9	8	9	6	6	4	3	1	8	5	4	2
**Pure technical efficiency**	**Mean**	0.882	0.017	0.903	0.005	0.866	−0.047	0.902	−0.020	0.914	0.001	0.884	−0.015	0.899	−0.014
	**Maximum**	1.000	0	1.000	0	1.000	0	1.000	0	1.000	0	1.000	0	1.000	0
	**Minimum**	0.538	0.008	0.636	0.015	0.595	−0.021	0.557	−0.059	0.638	−0.005	0.495	−0.151	0.673	−0.030
	**Standard deviation**	0.135	−0.012	0.100	−0.015	0.130	0.023	0.115	0.019	0.108	0.010	0.126	0.017	0.102	0.013
	**Hospital ranking**														
	**100%**	30	6	28	−6	27	−12	35	−4	38	6	34	4	31	2
	**80–99.9%**	31	−4	38	8	27	−3	32	−3	32	−7	29	−7	35	−9
	**60–79.9%**	20	−2	18	−2	29	14	16	6	14	1	19	1	18	7
	**<60%**	3	0	0	0	1	1	1	1	0	0	2	2	0	0
**Scale efficiency**	**Mean**	0.975	−0.049	0.907	−0.053	0.933	−0.024	0.951	−0.005	0.963	0.002	0.937	−0.035	0.941	−0.031
	**Maximum**	1.000	0	1.000	0	1.000	0	1.000	0	1.000	0	1.000	0	1.000	0
	**Minimum**	0.689	−0.239	0.339	−0.329	0.559	−0.104	0.564	−0.021	0.571	−0.121	0.335	−0.417	0.464	−0.195
	**Standard deviation**	0.048	0.056	0.131	0.07	0.086	0.023	0.080	0.012	0.075	0.012	0.105	0.061	0.086	0.031
	**Hospital ranking**														
	**100%**	29	−1	23	−3	24	−7	30	0	40	12	22	−3	22	−4
	**80–99.9%**	53	−7	46	−10	54	5	50	0	41	−12	53	−4	57	1
	**60–79.9%**	2	7	12	10	6	2	3	0	3	0	8	6	4	2
	**<60%**	0	1	3	3	0	0	1	0	0	0	1	1	1	1

#### Unchanged parts

The mean of scale efficiency remained larger than the means of technical efficiency and pure technical efficiency. This indicated that the environmental variable did not exert a significant influence on this phenomenon. In other words, scale efficiency was the major contributor to the hospital’s relative efficiency, implying a scope for expansion.

The maximal values for technical efficiency, pure technical efficiency and scale efficiency were all invariably 1.000. This indicated that for most hospitals, efficiencies remained optimal even after the environmental variable was introduced. This could be attributed to high levels of internal management and technologies of the evaluated hospitals. This finding can provide relevant government bodies and hospital management with more accurate decision making support.

The frequency distribution of scale efficiency remained the same for 2013 after adjustment of the input variable. Rather than the equality of “net” and “coarse” relative efficiencies, the scale efficiency that increased for some hospitals and decreased for others can explain the above result. This finding also demonstrates the ability of the environmental variable to correct the bias of evaluation results.

#### Changed parts

Technical efficiency and frequency distribution: the mean of technical efficiency varied for 2010–2016 (−0.063–−0.004). The minimum value of technical efficiency increased by 0.009 for 2013 and decreased for the remaining six years by a margin of 0.028−0.272. The frequency variation of technical efficiency showed that the number of hospitals in the 100% range decreased throughout the seven years except in 2010 and 2014, the number of hospitals in the 80−99.9% range decreased throughout, the number of hospitals in the 60−79.9% range increased throughout except for 2011and 2014, and the number of those in the < 60% range increased throughout. Therefore, it is viable to alleviate the high efficiency attributed to the congenial external environment. In addition, the results prove the significant influence of environmental factors on evaluation results, justifying the need for in-depth research.

Pure technical efficiency and frequency distribution: The mean of pure technical efficiency increased in 2010, 2011 and 2014 (0.017, 0.005,0.001) but decreased during the remaining five years (0.014−0.047). Its minimum value increased in 2010 and 2011 (0.008, 0.015) and decreased during the remaining four years (0.005−0.151). The number of hospitals with a high value of pure technical efficiency increased in 2010 and 2014−2016, but decreased during the remaining three years. The number of hospitals in the range of 80−99.9%, increased in 2011 and decreased for the remaining six years. The number of hospitals in the range < 60% increased for three years and remained the same in four years. Therefore, the fitting process was able to yield more objective efficiency and correct the bias of evaluation results.

The mean of scale efficiency varied for (−0.053–−0.002) but increased in 2014. The frequency distribution of hospital efficiency remained within a certain range. That is, the number of hospitals in the 100% range decreased except 2014 whereas those in the < 79.9% range increased. Hence, the high value of scale efficiency obtained in Stage 1 is susceptible to the environment and these effects need to be removed effectively during the efficiency evaluation.

The above analysis shows considerable variation of efficiency and its frequency distribution after the environmental variable is controlled and eliminated. Hence, we can conclude that the environmental factor has an impact on the evaluation results, and this observation should be considered during relative efficiency evaluation.

## Discussion

With increasing efforts for new medical reforms in China, research on efficiency evaluation for public hospitals is of increasing practical significance. This is because objective evaluations of efficiency can provide direct reference for government departments and hospital managers so facilitate pertinent efficiency measures. However, the relevant approaches and indices to select and how to control the impact factors have remained problematic. DEA is a multi-input and multi-output efficiency evaluation method, suitable for efficiency evaluation of public hospitals. However, past research results using this method have not been effectively applied to management decision making. One of the important reason for this is that the impact of the external environment on the efficiency evaluation results were not effectively controlled or eliminated. In other words, the precondition that the decision-making units should be as homogeneous as possible when using DEA for analysis cannot be ensured, resulting in questionable objectivity of the assessment results, which decreases their practical applicability.

The results of the present study indicate that during the Step 1 “initial” efficiency evaluation, the three relative efficiency measures of sample hospitals, namely technical efficiency (ranging from 0.860 to 0.890), pure technical efficiency (ranging from 0.882 to 0.922) and scale efficiency (ranging from 0.956 to 0.975), showed increasing trends, indicating that new medical reforms have played a significant role in improving the relative efficiencies of public hospitals and demonstrating the feasibility and effectiveness of the public hospital reform policy. However, the technical efficiency values of public hospitals in Chongqing were lower than those of other provinces. For example, from 2007 to 2013, the average technical efficiency values of public hospitals in Heilongjiang Province were 0. 960, 0. 930, 0. 960, 0. 950, 0.910, 0.920 and 0.890, respectively; the average pure technical efficiency values were 0.980, 0.970, 0.990, 0.980, 0.960, 0.970 and 0.960, respectively; the average scale efficiency values were 0.980, 0.960, 0.970, 0.970 0.940, 0.950 and 0.930 respectively [18]. From 2010 to 2014, the average technical efficiency values of public hospitals in Xinjiang were 1.000, 0.852, 0.953, 0.973 and 1.000, respectively; the average pure technical efficiency values were 1.000, 0.883, 0.970, 0.974 and 1.000, respectively; the average SE values were 1.000, 0.966, 0.982, 0.999 and 1.000 respectively [19]. In 2012, the average technical efficiency, pure technical efficiency and scale efficiency values of public hospitals in Tianjin were 0.893, 0.909 and 0.979, respectively [20]. These differences in evaluation results might be attributed to the index selection, hospital grade and other external factors

Second, it can be seen from the Tobit analysis in Step 2 that most of the eight external factors selected had an impact on the input slack variables, and we can draw the conclusion that the analysis of additional impact factors will have an impact on the efficiency evaluation results. Consequently, it is essential to use the Four-Stage-DEA analysis to evaluate the efficiency of public hospitals; otherwise, the homogeneity of the evaluated hospitals cannot be ensured in the DEA evaluation.

Third, it can be observed from the adjustment of original inputs in Step 3 that the adjusted average inputs are greater than the original inputs. In this way, all the evaluated decision-making units are placed in the worst external environment, thereby placing the sample hospitals on the same external platform to allow more efficient, objective and scientific efficiency evaluation results.

Fourth, it can be seen in Step 4 that during the Four-Stage-DEA analysis, the results obtained from Step 4 were quite different from those obtained from Step 1. The Four-Stage-DEA analysis is different from other commonly-used models such as DEA-CCR, DEA-BCC and Malmquist or models of decreasing indices. One of the characteristics of the present study is that the number of relatively-efficient hospitals almost changed and the mean, median and minimum scale efficiencies of the sample hospitals changed too in different years. These phenomena show that the new medical reform plays a significant role in improving the efficiencies of public hospitals; however, it is essential to eliminate and control the impact factors.

## Conclusions

The Four-Stage-DEA method was adopted in this paper to quantitatively evaluate the technical, pure technical and scale efficiencies achieved by 84 public hospitals in Chongqing during 2010–2016. To the best of our knowledge, this method has not previously been applied elsewhere in the medical field in China. The influence of environmental factors on the evaluation result was controlled and eliminated by comparing relative efficiencies with and without considering the environmental factors. The adopted Four-Stage-DEA method is different from the Three-Stage-DEA method and the traditional DEA model. Conclusions from this study are summarized as follows.

1. The study on factors is unique: Although the Tobit regression analysis has been performed in previous studies where the input/output metrics were compared with the efficiency evaluation results, the unique feature of the present study was the addition of environmental variable into the analysis, and the emphasis on the homogeneity of evaluated hospitals during DEA. We found that the direction and amplitude of the influence that a specific environmental factor has on a specific input slack variable varied across years. Therefore, an appropriate and controllable range has not yet been identified in the context of unbalanced deployment of medical resources and it should focus on in the future research and management practices.
2. The external environment has an impact on evaluation result of relative efficiency: The mean of scale efficiency was consistently higher than those of the two other efficiencies throughout the seven years, even though it decreased during most years. This implies that traditional studies using conventional models have overestimated relative efficiencies of hospitals, thereby limiting their usefulness for decision makers.
3. The scope for relative efficiency improvement is high: For each of the three relative efficiencies, the mean was high and over 0.800[21] at Stages 1 and 4. However, there is scope for improvement for two thirds of the hospitals. For some hospitals, the efficiencies were consistently below 60% across the seven years after the addition of environmental variables (e.g., H7). These inefficiencies should therefore be treated very carefully.
4. More attention should be paid to this problem in future studies: Efficiency evaluation is an important content of the new medical reform and is very necessary in solving the problem of limited and unbalanced medical resources because efficiency evaluation is needed in the supply-side structural reform in the medical and health fields. The idea and method for evaluation of relative efficiency should be put into practice. The ultimate aim of the present study is to put our conclusions into practice. To this end, the metrics and their weights need to be appropriately determined and the factors need to be evaluated. The Four-Stage-DEA method, which was adopted in the present study to control environmental variables, can be combined with DEA-Bootstrap to control random error and improve reliability of the evaluation results.

## Supporting information

S1 Table Relevant data underlying the findings described in manuscript at Stage 1 from 2010 to 2016. (XLSX)

S2 Table Relevant data underlying the findings described in manuscript at Stage 2 from 2010 to 2016. (CSV)

S3 Table Relevant data underlying the findings described in manuscript at Stage 3 from 2010 to 2016. (CSV)

S4 Table Relevant data underlying the findings described in manuscript at Stage 4 from 2010 to 2016. (CSV)
